# Supplementary material for: Evaluation of plasma tau and neurofilament light chain biomarkers in a 12-year clinical cohort of human prion diseases
Source: Mol Psychiatry. 2021 Mar 5;26(10):5955–66. doi: 10.1038/s41380-021-01045-w (PMC8758487; doi:10.1038/s41380-021-01045-w)
Supplement: Supplementary file 1 — Supplementary Material [file 41380_2021_1045_MOESM1_ESM.docx]

**Supplementary Material**

**Evaluation of plasma tau and neurofilament light chain biomarkers in a 12-year clinical cohort of human prion diseases**

Andrew GB Thompson^1^, Prodromos Anastasiadis^1^, Ronald Druyeh^1^, Ines Whitworth^1^, Akin Nihat^1,2^, Tze How Mok^1,2^, Peter Rudge^1,2^, Jonathan DF Wadsworth^1^, Jonathan Rohrer^3^, Jonathan Schott ^3^, Amanda Heslegrave^4^, Henrik Zetterberg^4,5,6^, John Collinge^1,2^, Graham S Jackson^1^, Simon Mead^1,2^.

Contents

[Supplementary Methods: Quality control of Simoa analysis 3](#_Toc63154651)

[Supplementary Results: Predicting clinical progression in sCJD using baseline biomarker results 3](#_Toc63154652)

[Supplementary Table 1. Summary statistics for plasma tau and NfL in diagnostic subgroups of the CJD mimics included in the study. 5](#_Toc63154653)

[Supplementary Table 2: Linear regression for baseline log-tau and log-NfL in sCJD, with age, gender, PRNP codon 129 genotype, MRC Score and MRC Slope as covariates 6](#_Toc63154654)

[Supplementary Table 3A: Linear mixed effects model for change in log-tau over time, with random effects by individual 7](#_Toc63154655)

[Supplementary Table 3B: Linear mixed effects model for change in Tau (log-tau) with change in MRC Scale, with random effects by individual 8](#_Toc63154656)

[Supplementary Table 3C: Linear mixed effects model for change in NfL (log-NfL) over time, with random effects by individual 9](#_Toc63154657)

[Supplementary Table 3D: Linear mixed effects model for change in NfL (log-NfL) with change in MRC Score, with random effects by individual 10](#_Toc63154658)

[Supplementary Table 4. PrP^Sc^ type and plasma tau and NfL levels 11](#_Toc63154659)

[Supplementary Figure 1. Distribution of rate of change in plasma tau and NfL between all serial sample pairs in sCJD 12](#_Toc63154660)

[Supplementary Figure 2. Comparing plasma tau and plasma NfL (both shown on log scales) between *PRNP* mutations in symptomatic IPD 13](#_Toc63154661)

[Supplementary Figure 3. Relationship of plasma tau and NfL to number of days from death in symptomatic IPD, including both cross-sectional and longitudinal datasets 14](#_Toc63154662)

[Supplementary Figure 4. Plasma NfL concentrations plotted against age at time of sampling for controls and asymptomatic at-risk individuals 15](#_Toc63154663)

[Supplementary Figure 5. Relationship of plasma tau and NfL to number of days from death in iCJD and vCJD, including both cross-sectional and longitudinal datasets 17](#_Toc63154664)

[References 18](#_Toc63154665)

# Supplementary Methods: Quality control of Simoa analysis

For plasma tau, 87% of the tested samples produced two technically satisfactory results with a coefficient of variation (CV) < 15% on their first assay run. Samples with a higher CV, or for which at least one of the duplicate assays did not produce a result (due to technical issues with the analyser), were re-tested. Following this, the mean CV for duplicate results was 6.3%. 16 samples still had only one technically satisfactory result available – as CVs were low, these single results were also included. The CVs for high and low concentration inter-plate controls across 10 plates tested were both <15%.

For plasma NfL, 95% of the tested samples produced two technically satisfactory results with a CV < 15% on their first assay run. Samples with a higher CV, or for which at least one of the duplicate assays did not produce a result (due to technical issues with the analyser), were re-tested. Following this, the mean CV for duplicate results was 4.8%. 5 samples still had only one technically satisfactory result available – as the CVs were low these single results were also included. The CVs for high and low concentration inter-plate controls across 10 plates tested were both <20%.

One sample produced results for both tau and NfL that were well below the physiological range seen in any healthy controls or patients, and so was excluded on the basis that this was likely to represent a pre-analytical issue (e.g. accidental dilution of sample).

In total, after quality control, 814 samples were included in the data analysis for plasma tau, and 833 samples were included for plasma NfL.

# Supplementary Results: Predicting clinical progression in sCJD using baseline biomarker results

We explored whether baseline assessment of plasma tau or NfL concentration could help to predict subsequent rate of clinical disease progression in sCJD patients, and thereby have potential value for stratifying patients at enrolment to clinical trials. We added these baseline biomarker measures to the previously published linear mixed models of decline in MRC Scale^1^, for those patients that had a sample tested in this study which was taken prior to, or at the time of, their enrolment to the clinical Cohort study: 98 patients in total. Models using untransformed biomarker concentration values, log-transformed values, and with truncation of outliers (values > 95^th^ percentile set at the 95^th^ percentile value, to prevent over-fitting to extreme numerical outliers), and including fixed effects for age and *PRNP* codon 129 genotype, were compared using Akaike Information Criterion (AIC).

For NfL, the best fitting model was that including log-transformed plasma NfL concentration (AIC=4767.4, compared with AIC=4781.2 for the model without baseline biomarkers as previously published). This showed an independent effect of MRC Scale on intercept (p<0.001), with higher baseline log plasma NfL predicting lower MRC Scale at enrolment. However, there was no independent effect on subsequent rate of change in MRC Scale over time. As MRC Scale at enrolment can be directly assessed in all patients, predicting this does not provide any additional clinical utility.

For tau, the best fitting model (AIC=4776.3, compared with AIC=4781.2 for the standard model) was that including untransformed plasma tau concentration. This showed an effect of baseline plasma tau on subsequent rate of decline in MRC Scale (p=0.008), but this appeared to be driven by outliers (a small number of patients with extremely high baseline tau and very rapid clinical decline), as it did not persist after truncation of plasma tau values > the 95^th^ percentile (p=0.659). Exploring these models appeared to show a differential effect of baseline plasma tau on rate of decline in MRC Scale between different *PRNP* codon 129 genotypes. We therefore also explored modelling baseline plasma tau as a predictor separately within each codon 129 genotype. Within the MM group (31 patients), higher baseline concentrations of plasma tau were associated with more rapid subsequent decline in MRC Scale over time (p<0.001), and this persisted after truncation of values > 95^th^ percentile (p<0.001), and was also present for log-transformed plasma tau concentration (p<0.001). In contrast there was no effect of baseline plasma tau on subsequent rate of clinical decline in the models for MV (n=43) or VV (n=24) patients. There may therefore be some additional predictive value if baseline plasma tau is included in predictive models in a specific subset of patients, but the overall benefit from adding this into existing models appears to be small.

# Supplementary Table 1. Summary statistics for plasma tau and NfL in diagnostic subgroups of the CJD mimics included in the study.

| **Subgroups of CJD mimics** | **Individuals** | **Plasma tau (pg/mL)** | | | **Plasma NfL (pg/mL)** | | |
| --- | --- | --- | --- | --- | --- | --- | --- |
|  | n | **Min** | **Median** | Max | Min | **Median** | Max |
|  |  |  |  |  |  |  |  |
| Other neurodegenerative conditions | 15 | 0.60 | **1.96** | 3.57 | 9.27 | **53.90** | 593.34 |
| Cerebrovascular disease | 2 | 6.08 | **7.49** | 8.90 | 36.40 | **100.76** | 165.12 |
| Autoimmune Encephalitis | 4 | 1.84 | **3.56** | 3.79 | 22.70 | **51.95** | 141.11 |
| CNS Lymphoma | 1 | - | **3.29** | **-** |  | **353.44** |  |
| Hepatic encephalopathy | 1 | - | **5.24** | - |  | **9.09** |  |
| Progressive Multifocal Leucoencephalopathy | 1 | - | **10.19** | **-** |  | **232.18** |  |
|  |  |  |  |  |  |  |  |
| **All CJD mimics** | **24** | 0.60 | **3.04** | 10.19 | 9.09 | **53.90** | 593.34 |
|  |  |  |  |  |  |  |  |

# Supplementary Table 2: Linear regression for baseline log-tau and log-NfL in sCJD, with age, gender, PRNP codon 129 genotype, MRC Score and MRC Slope as covariates

| **Log -tau** |  | | | | | | |
| --- | --- | --- | --- | --- | --- | --- | --- |
|  |  | **Coef.** | **SE** | **t** | **p value** | **95% CI (Coef.)** | |
|  | Age | -0.0006511 | 0.0024197 | -0.27 | 0.788 | -0.0054325 | 0.0041304 |
|  | Gender | -0.0707005 | 0.0595624 | -1.19 | 0.237 | -0.1883966 | 0.0469956 |
|  | Codon 129 | -0.0425298 | 0.0457946 | -0.93 | 0.355 | -0.1330206 | 0.047961 |
|  | MRC Score | -0.0128264 | 0.0064227 | -2 | 0.048 | -0.0255177 | -0.000135 |
|  | **MRC Slope** | **0.2777325** | **0.0388402** | **7.15** | **<0.001** | **0.2009838** | **0.3544812** |
|  | **Constant** | **0.8041434** | **0.182551** | **4.41** | **<0.001** | **0.4434202** | **1.164867** |
|  | **Overall R-squared = 0.358** | | |  |  |  |  |
|  |  |  |  |  |  |  |  |
| **Log-NfL** |  | | | | | | |
|  |  | **Coef.** | **SE** | **t** | **p value** | **95% CI (Coef.)** | |
|  | Age | 0.0057589 | 0.0022414 | 2.57 | 0.011 | 0.0013298 | 0.010188 |
|  | Gender | 0.0625685 | 0.0552005 | 1.13 | 0.259 | -0.0465084 | 0.1716454 |
|  | Codon 129 | 0.0959208 | 0.0425146 | 2.26 | 0.026 | 0.0119114 | 0.1799303 |
|  | **MRC Score** | **-0.034434** | **0.0059151** | **-5.82** | **<0.001** | **-0.0461223** | **-0.0227458** |
|  | MRC Slope | 0.0642922 | 0.0360644 | 1.78 | 0.077 | -0.0069716 | 0.135556 |
|  | **Constant** | **1.743891** | **0.1688592** | **10.33** | **<0.001** | **1.410223** | **2.077559** |
|  | **Overall R-Squared = 0.359** | | |  |  |  |  |

# Supplementary Table 3A: Linear mixed effects model for change in log-tau over time, with random effects by individual

|  | Mixed-effects REML regression | | | | | | | | | | Number of samples = 101 | | | | | | | | | | | | | | | | | | | | | | | | | | | | | | | | | |  | |  | | | | | | | | | |  | | | | | | | | | | | |  |  |  |  |  |  |  |  |  |
| --- | --- | --- | --- | --- | --- | --- | --- | --- | --- | --- | --- | --- | --- | --- | --- | --- | --- | --- | --- | --- | --- | --- | --- | --- | --- | --- | --- | --- | --- | --- | --- | --- | --- | --- | --- | --- | --- | --- | --- | --- | --- | --- | --- | --- | --- | --- | --- | --- | --- | --- | --- | --- | --- | --- | --- | --- | --- | --- | --- | --- | --- | --- | --- | --- | --- | --- | --- | --- | --- | --- | --- | --- | --- | --- | --- | --- | --- |
|  |  | | | | | |  | | | | Number of individuals = 41 | | | | | | | | | | | | | | | | | | | | | | | | | | | | | | | | | |  | |  | | | | | | | | | |  | | | | | | | | | | | |  |  |  |  |  |  |  |  |  |
|  | Wald Chi2(6) = 14.60 | | | | | |  | | | |  | | | | | | | | | |  | | | | | | | | | | | | |  | | | | | | | | | | |  | |  | | | | | | | | | |  | | | | | | | | | | | |  |  |  |  |  |  |  |  |  |
|  | Log restricted-likelihood = -39.616441 | | | | | | | | | | Prob > Chi2 = 0.0236 | | | | | | | | | | | | | | | | | | | | | | |  | | | | | | | | | | |  | |  | | | | | | | | | |  | | | | | | | | | | | |  |  |  |  |  |  |  |  |  |
|  | **Fixed effects** | | | | | | Coef. | | | | | SE | | | | | | | | | z | | | | | | | | | | | | | p value | | 95% CI (Coef.) | | | | | | | | | | | | | | | | | | | | | | |  | | | | | | | | | | |  |  |  |  |  |  |  |  |
|  | Time (days from death) | | | | | | -0.0001633 | | | | | 0.0001181 | | | | | | | | | -1.38 | | | | | | | | | | | | | 0.167 | | -0.0003948 | | | | | | | | | | | | 0.0000683 | | | | | | | | | | |  | | | | | | | | | | |  |  |  |  |  |  |  |  |
|  | Age | | |  | | | 0.0037123 | | | | | 0.0048443 | | | | | | | | | 0.77 | | | | | | | | | | | | | 0.443 | | -0.0057823 | | | | | | | | | | | | 0.0132069 | | | | | | | | | | |  | | | | | | | | | | |  |  |  |  |  |  |  |  |
|  | *PRNP* codon 129 genotype (relative to MV) effect on intercept | | | | | | | | | | | | | | | | | | | | | | | | | |  | | | | | | | | |  | | | | | | | | | | | |  | | | | | | | | | | |  | | | | | | | | | | |  |  |  |  |  |  |  |  |
|  |  | | | MM | | | -0.0254731 | | | | | 0.2012527 | | | | | | | | | -0.13 | | | | | | | | | | | | | 0.899 | | -0.4199211 | | | | | | | | | | | | 0.3689748 | | | | | | | | | | |  | | | | | | | | | | |  |  |  |  |  |  |  |  |
|  |  | | | VV | | | -0.0233208 | | | | | 0.1565643 | | | | | | | | | -0.15 | | | | | | | | | | | | | 0.882 | | -0.3301812 | | | | | | | | | | | | 0.2835396 | | | | | | | | | | |  | | | | | | | | | | |  |  |  |  |  |  |  |  |
|  | *PRNP* codon 129 genotype (relative to MV) effect on slope (log-tau vs time) | | | | | | | | | | | | | | | | | | | | | | | | | | | | | | | | | | |  | | | | | | | | | | | |  | | | | | | | | | | |  | | | | | | | | | | |  |  |  |  |  |  |  |  |
|  | 1 | | | MM | | | 0.0004924 | | | | | 0.000468 | | | | | | | | | 1.05 | | | | | | | | | | | | | 0.293 | | -0.0004248 | | | | | | | | | | | | 0.0014096 | | | | | | | | | | |  | | | | | | | | | | |  |  |  |  |  |  |  |  |
|  | 3 | | | VV | | | 0.0032901 | | | | | 0.0013407 | | | | | | | | | 2.45 | | | | | | | | | | | | | 0.014 | | 0.0006624 | | | | | | | | | | | | 0.0059178 | | | | | | | | | | |  | | | | | | | | | | |  |  |  |  |  |  |  |  |
|  | Constant | | |  | | | 0.3564546 | | | | | 0.3058501 | | | | | | | | | 1.17 | | | | | | | | | | | | | 0.244 | | -0.2430005 | | | | | | | | | | | | 0.9559097 | | | | | | | | | | |  | | | | | | | | | | |  |  |  |  |  |  |  |  |
|  | **Random effects (by individual)** | | | | | | | | | |  | | | | | | | | | |  | | | | | | | | | | | | |  | | | | | | | | | | |  | |  | | | | | | | | | |  | | | | | | | | | | | |  |  |  |  |  |  |  |  |  |
|  | Parameters | | |  | | | Estimate | | | | SE | | | | | | | | | | 95% CI (Estimate) | | | | | | | | | | | | | | | | | | | | | | | |  | |  | | | | | | | | | |  | | | | | | | | | | | |  |  |  |  |  |  |  |  |  |
|  | SD (Constant) | | | | | | 0.2124896 | | | | 0.0472058 | | | | | | | | | | 0.1374797 | | | | | | | | | | | | | 0.3284255 | | | | | | | | | | |  | |  | | | | | | | | | |  | | | | | | | | | | | |  |  |  |  |  |  |  |  |  |
|  | SD (Days from death) | | | | | | 7.79E-11 | | | | . | | | | | | | | | | . | | | | | | | | | | | | | . | | | | | | | | | | |  | |  | | | | | | | | | |  | | | | | | | | | | | |  |  |  |  |  |  |  |  |  |
|  | Residuals: | | | Independent | | | | | | |  | | | | | | | | | |  | | | | | | | | | | | | |  | | | | | | | | | | |  | |  | | | | | | | | | |  | | | | | | | | | | | |  |  |  |  |  |  |  |  |  |
|  | By *PRNP* codon 129 genotype | | | | | | | | | |  | | | | | | | | | |  | | | | | | | | | | | | |  | | | | | | | | | | |  | |  | | | | | | | | | |  | | | | | | | | | | | |  |  |  |  |  |  |  |  |  |
|  | MM | | | sd(e) | | | 0.3340261 | | | | 0.1299548 | | | | | | | | | | 0.1558172 | | | | | | | | | | | | | 0.7160533 | | | | | | | | | | |  | |  | | | | | | | | | |  | | | | | | | | | | | |  |  |  |  |  |  |  |  |  |
|  | MV | | | sd(e) | | | 0.197673 | | | | 0.0211525 | | | | | | | | | | 0.1602739 | | | | | | | | | | | | | 0.2437992 | | | | | | | | | | |  | |  | | | | | | | | | |  | | | | | | | | | | | |  |  |  |  |  |  |  |  |  |
|  | VV | | | sd(e) | | | 0.2275296 | | | | 0.0508104 | | | | | | | | | | 0.1468768 | | | | | | | | | | | | | 0.3524704 | | | | | | | | | | |  | |  | | | | | | | | | |  | | | | | | | | | | | |  |  |  |  |  |  |  |  |  |
|  |  | | |  | | |  | | | |  | | | | | | | | | |  | | | | | | | | | | | | |  | | | | | | | | | | |  | |  | | | | | | | | | |  | | | | | | | | | | | |  |  |  |  |  |  |  |  |  |
|  | LR test vs. linear regression: Chi2(4) = 21.66 | | | | | | | | | | | | | | | | | | | | Prob > Chi2 = 0.0002 | | | | | | | | | | | | | | | | | | | | | | | |  | |  | | | | | | | | | |  | | | | | | | | | | | |  |  |  |  |  |  |  |  |  |
| Supplementary Table 3B: Linear mixed effects model for change in Tau (log-tau) with change in MRC Scale, with random effects by individual | | | | | | | | | | | | | | | | | | | | | | | | | | | | | | | | | | | | | | | | | | | | | | | | | | | | | | | | | | | | | | | | | | | | |  |  |  |  |  |  |  |  |  |
|  | Mixed-effects REML regression | | | | | | | | | | Number of samples = 91 | | | | | | | | | | | | | | | | | | | | |  | | | | | | | | | |  | | | | | | | | | |  | | | | | | | |  | | | | | | | | | | | | | |  |  |  |  |
|  | Group variable: cohortid | | | | | |  | | | | Number of individuals = 40 | | | | | | | | | | | | | | | | | | | | | | | | | | | | | | |  | | | | | | | | | |  | | | | | | | |  | | | | | | | | | | | | | |  |  |  |  |
|  | Wald Chi2(6) = 14.80 | | | | | |  | | | |  | | | | | | | | | |  | | | | | | | | | | |  | | | | | | | | | |  | | | | | | | | | |  | | | | | | | |  | | | | | | | | | | | | | |  |  |  |  |
|  | Log restricted-likelihood = -29.306071 | | | | | | | | | | Prob > Chi2 = 0.0218 | | | | | | | | | | | | | | | | | | | | |  | | | | | | | | | |  | | | | | | | | | |  | | | | | | | |  | | | | | | | | | | | | | |  |  |  |  |
|  | **Fixed effects** | | | | | | Coef. | | | | | | | SE | | | | | | | | z | | | | | | | p value | | | | | | | | | 95% CI (Coef.) | | | | | | | | | | | | | | | | | | | | | | | |  | | | | | | | | | | | | |  |  |  |
|  | MRC Score | | |  | | | -0.0159666 | | | | | | | 0.0066046 | | | | | | | | -2.42 | | | | | | | 0.016 | | | | | | | | | -0.0289114 | | | | | | | | | | | | -0.0030218 | | | | | | | | | | | |  | | | | | | | | | | | | |  |  |  |
|  | Age | | |  | | | -0.0027268 | | | | | | | 0.0043042 | | | | | | | | -0.63 | | | | | | | 0.526 | | | | | | | | | -0.0111629 | | | | | | | | | | | | 0.0057093 | | | | | | | | | | | |  | | | | | | | | | | | | |  |  |  |
|  | *PRNP* codon 129 genotype (relative to MV) effect on intercept | | | | | | | | | | | | | | | | | | | | | | | | | | | |  | | | | | | | | |  | | | | | | | | | | | |  | | | | | | | | | | | |  | | | | | | | | | | | | |  |  |  |
|  |  | | | MM | | | 0.3154141 | | | | | | | 0.3622733 | | | | | | | | 0.87 | | | | | | | 0.384 | | | | | | | | | -0.3946285 | | | | | | | | | | | | 1.025457 | | | | | | | | | | | |  | | | | | | | | | | | | |  |  |  |
|  |  | | | VV | | | 0.2756725 | | | | | | | 0.1716547 | | | | | | | | 1.61 | | | | | | | 0.108 | | | | | | | | | -0.0607645 | | | | | | | | | | | | 0.6121095 | | | | | | | | | | | |  | | | | | | | | | | | | |  |  |  |
|  | *PRNP* codon 129 genotype (relative to MV) effect on slope (log-tau vs time) | | | | | | | | | | | | | | | | | | | | | | | | | | | | | | | | | | | | |  | | | | | | | | | | | |  | | | | | | | | | | | |  | | | | | | | | | | | | |  |  |  |
|  | 1 | | | MM | | | -0.0263727 | | | | | | | 0.0372293 | | | | | | | | -0.71 | | | | | | | 0.479 | | | | | | | | | -0.0993407 | | | | | | | | | | | | 0.0465953 | | | | | | | | | | | |  | | | | | | | | | | | | |  |  |  |
|  | 3 | | | VV | | | -0.0028959 | | | | | | | 0.0184395 | | | | | | | | -0.16 | | | | | | | 0.875 | | | | | | | | | -0.0390367 | | | | | | | | | | | | 0.0332448 | | | | | | | | | | | |  | | | | | | | | | | | | |  |  |  |
|  | Constant | | |  | | | 0.8436671 | | | | | | | 0.276107 | | | | | | | | 3.06 | | | | | | | 0.002 | | | | | | | | | 0.3025074 | | | | | | | | | | | | 1.384827 | | | | | | | | | | | |  | | | | | | | | | | | | |  |  |  |
|  | **Random effects (by individual)** | | | | | | | | | |  | | | | | | | | | |  | | | | | | | | | | | | |  | | | | | | | | | | |  | |  | | | | | | | | | |  | | | | | | | | | | | |  |  |  |  |  |  |  |  |  |
|  | Parameters | | |  | | | Estimate | | | | SE | | | | | | | | | | 95% CI (Estimate) | | | | | | | | | | | | | | | | | | | | | | | |  | |  | | | | | | | | | |  | | | | | | | | | | | |  |  |  |  |  |  |  |  |  |
|  | SD (Constant) | | | | | | 0.2040276 | | | | 0.0477484 | | | | | | | | | | 0.1289683 | | | | | | | | | | | | | 0.3227713 | | | | | | | | | | |  | |  | | | | | | | | | |  | | | | | | | | | | | |  |  |  |  |  |  |  |  |  |
|  | SD (MRC Score) | | | | | | 0.0043681 | | | | 0.0202764 | | | | | | | | | | 4.89E-07 | | | | | | | | | | | | | 39.03827 | | | | | | | | | | |  | |  | | | | | | | | | |  | | | | | | | | | | | |  |  |  |  |  |  |  |  |  |
|  | Residuals: | | | Independent | | | | | | |  | | | | | | | | | |  | | | | | | | | | | | | |  | | | | | | | | | | |  | |  | | | | | | | | | |  | | | | | | | | | | | |  |  |  |  |  |  |  |  |  |
|  | By PRNP codon 129 genotype | | | | | | | | | |  | | | | | | | | | |  | | | | | | | | | | | | |  | | | | | | | | | | |  | |  | | | | | | | | | |  | | | | | | | | | | | |  |  |  |  |  |  |  |  |  |
|  | MM | | | sd(e) | | | 0.4906119 | | | | 0.1580161 | | | | | | | | | | 0.2609657 | | | | | | | | | | | | | 0.9223436 | | | | | | | | | | |  | |  | | | | | | | | | |  | | | | | | | | | | | |  |  |  |  |  |  |  |  |  |
|  | MV | | | sd(e) | | | 0.1970122 | | | | 0.0249731 | | | | | | | | | | 0.1536723 | | | | | | | | | | | | | 0.2525751 | | | | | | | | | | |  | |  | | | | | | | | | |  | | | | | | | | | | | |  |  |  |  |  |  |  |  |  |
|  | VV | | | sd(e) | | | 0.2395811 | | | | 0.0605531 | | | | | | | | | | 0.1459872 | | | | | | | | | | | | | 0.3931789 | | | | | | | | | | |  | |  | | | | | | | | | |  | | | | | | | | | | | |  |  |  |  |  |  |  |  |  |
|  | LR test vs. linear regression: chi2(4) = 19.62 | | | | | | | | | | | | | | | | | | | | Prob > Chi2 = 0.0006 | | | | | | | | | | | | | | | | | | | | | | | |  | |  | | | | | | | | | |  | | | | | | | | | | | |  |  |  |  |  |  |  |  |  |
| Supplementary Table 3C: Linear mixed effects model for change in NfL (log-NfL) over time, with random effects by individual | | | | | | | | | | | | | | | | | | | | | | | | | | | | | | | | | | | | | | | | | | | | | | | | | | | | | | | | |  | | | | | | | | | | | |  |  |  |  |  |  |  |  |  |
|  | Mixed-effects REML regression | | | | | | | | | | Number of samples = 108 | | | | | | | | | | | | | | | | | | | | | | | | | | | | | | | | |  | | |  | | | | | | | | | |  | | | | | | | | | | | |  |  |  |  |  |  |  |  |  |
|  | Group variable: cohortid | | | | | |  | | | | Number of individuals = 44 | | | | | | | | | | | | | | | | | | | | | | | | | | | | | | | | |  | | |  | | | | | | | | | |  | | | | | | | | | | | |  |  |  |  |  |  |  |  |  |
|  | Wald Chi2(6) = 34.68 | | | | | |  | | | |  | | | | | | | |  | | | | | | | | | | | | | |  | | | | | | | | | | |  | | |  | | | | | | | | | |  | | | | | | | | | | | |  |  |  |  |  |  |  |  |  |
|  | Log restricted-likelihood = -21.849083 | | | | | | | | | | Prob > Chi2 = 0.0000 | | | | | | | | | | | | | | | | | | | | | |  | | | | | | | | | | |  | | |  | | | | | | | | | |  | | | | | | | | | | | |  |  |  |  |  |  |  |  |  |
|  | **Fixed effects** | | | | | | Coef. | | | | | | SE | | | | | | | z | | | | | | | | p value | | | | | | | | | 95% CI (Coef.) | | | | | | | | | | | | | | | | | | | | | | | |  | | | | | | | | | | |  |  |  |  |  |  |
|  | Time (days from death) | | | | | | -0.0008608 | | | | | | 0.0002032 | | | | | | | -4.24 | | | | | | | | 0 | | | | | | | | | -0.0012592 | | | | | | | | | | | | -0.0004625 | | | | | | | | | | | |  |  |  |  |  |  |  |  |  |  |  |  |  |  |  |  |  |
|  | Age | | |  | | | -0.0123781 | | | | | | 0.0050515 | | | | | | | -2.45 | | | | | | | | 0.014 | | | | | | | | | -0.0222789 | | | | | | | | | | | | -0.0024773 | | | | | | | | | | | |  | | | | | | | | | | |  |  |  |  |  |  |
|  | *PRNP* codon 129 genotype (relative to MV) effect on intercept | | | | | | | | | | | | | | | | | | | | | | | | | | |  | | | | | | | | |  | | | | | | | | | | | |  | | | | | | | | | | | |  | | | | | | | | | | |  |  |  |  |  |  |
|  |  | | | MM | | | -0.1690149 | | | | | | 0.1845158 | | | | | | | -0.92 | | | | | | | | 0.36 | | | | | | | | | -0.5306592 | | | | | | | | | | | | 0.1926294 | | | | | | | | | | | |  | | | | | | | | | | |  |  |  |  |  |  |
|  |  | | | VV | | | 0.2321624 | | | | | | 0.1354934 | | | | | | | 1.71 | | | | | | | | 0.087 | | | | | | | | | -0.0333998 | | | | | | | | | | | | 0.4977245 | | | | | | | | | | | |  | | | | | | | | | | |  |  |  |  |  |  |
|  | *PRNP* codon 129 genotype (relative to MV) effect on slope (log-tau vs time) | | | | | | | | | | | | | | | | | | | | | | | | | | | | | | | | | | | |  | | | | | | | | | | | |  | | | | | | | | | | | |  | | | | | | | | | | |  |  |  |  |  |  |
|  | 1 | | | MM | | | 0.0002357 | | | | | | 0.0005435 | | | | | | | 0.43 | | | | | | | | 0.665 | | | | | | | | | -0.0008296 | | | | | | | | | | | | 0.001301 | | | | | | | | | | | |  | | | | | | | | | | |  |  |  |  |  |  |
|  | 3 | | | VV | | | -0.0027947 | | | | | | 0.0010511 | | | | | | | -2.66 | | | | | | | | 0.008 | | | | | | | | | -0.0048548 | | | | | | | | | | | | -0.0007346 | | | | | | | | | | | |  | | | | | | | | | | |  |  |  |  |  |  |
|  | Constant | | |  | | | 3.106646 | | | | | | 0.3247861 | | | | | | | 9.57 | | | | | | | | 0 | | | | | | | | | 2.470077 | | | | | | | | | | | | 3.743215 | | | | | | | | | | | |  |  |  |  |  |  |  |  |  |  |  |  |  |  |  |  |  |
|  | **Random effects (by individual)** | | | | | | | | | |  | | | | | | | |  | | | | | | | | | | | | | |  | | | | | | | | | | |  | | |  | | | | | | | | | |  | | | | | | | | | | | |  |  |  |  |  |  |  |  |  |
|  | Parameters | | |  | | | Estimate | | | | SE | | | | | | | | 95% CI (Estimate) | | | | | | | | | | | | | | | | | | | | | | | | |  | | |  | | | | | | | | | |  | | | | | | | | | | | |  |  |  |  |  |  |  |  |  |
|  | SD (Constant) | | | | | | 0.2500583 | | | | 0.0386523 | | | | | | | | 0.1847007 | | | | | | | | | | | | | | 0.3385432 | | | | | | | | | | |  | | |  | | | | | | | | | |  | | | | | | | | | | | |  |  |  |  |  |  |  |  |  |
|  | SD (Days from death) | | | | | | 0.0004722 | | | | 0.0001807 | | | | | | | | 0.0002231 | | | | | | | | | | | | | | 0.0009996 | | | | | | | | | | |  | | |  | | | | | | | | | |  | | | | | | | | | | | |  |  |  |  |  |  |  |  |  |
|  | Residuals: | | | Independent | | | | | | |  | | | | | | | |  | | | | | | | | | | | | | |  | | | | | | | | | | |  | | |  | | | | | | | | | |  | | | | | | | | | | | |  |  |  |  |  |  |  |  |  |
|  | By PRNP codon 129 genotype | | | | | | | | | |  | | | | | | | |  | | | | | | | | | | | | | |  | | | | | | | | | | |  | | |  | | | | | | | | | |  | | | | | | | | | | | |  |  |  |  |  |  |  |  |  |
|  | MM | | | sd(e) | | | 0.2224403 | | | | 0.062041 | | | | | | | | 0.1287669 | | | | | | | | | | | | | | 0.3842577 | | | | | | | | | | |  | | |  | | | | | | | | | |  | | | | | | | | | | | |  |  |  |  |  |  |  |  |  |
|  | MV | | | sd(e) | | | 0.1315952 | | | | 0.0174293 | | | | | | | | 0.1015083 | | | | | | | | | | | | | | 0.1705998 | | | | | | | | | | |  | | |  | | | | | | | | | |  | | | | | | | | | | | |  |  |  |  |  |  |  |  |  |
|  | VV | | | sd(e) | | | 0.1403208 | | | | 0.031528 | | | | | | | | 0.0903379 | | | | | | | | | | | | | | 0.2179588 | | | | | | | | | | |  | | |  | | | | | | | | | |  | | | | | | | | | | | |  |  |  |  |  |  |  |  |  |
|  | LR test vs. linear regression: Chi2(4) = 41.98 | | | | | | | | | | | | | | | | | | Prob > Chi2 = 0.0000 | | | | | | | | | | | | | | | | | | | | | | | | |  | | |  | | | | | | | | | |  | | | | | | | | | | | |  |  |  |  |  |  |  |  |  |
| Supplementary Table 3D: Linear mixed effects model for change in NfL (log-NfL) with change in MRC Score, with random effects by individual | | | | | | | | | | | | | | | | | | | | | | | | | | | | | | | | | | | | | | | | | | | | | | | | | | | | | | | | | | | | | | | | | | | | |  |  |  |  |  |  |  |  |  |
|  | Mixed-effects REML regression | | | | | | | | | Number of samples = 90 | | | | | | | | | | | | | | | | | | | | | | |  | | | | | | | | | |  | | | | | | | | | |  | | | | | | | | | | |  | | | | | | | | |  | | | | |
|  | Group variable: cohortid | | | | | | | | | Number of individuals = 40 | | | | | | | | | | | | | | | | | | | | | | | | | | | | | |  | | | | | | | | | | |  | | | | | | | | | | | |  | | | | | | | |  | | | | | |  |
|  | Wald Chi2(6) = 30.58 | | | | | | | | |  | | | | | | | |  | | | | | | |  | | | | | | | | | |  | | | | | | | | | | |  | | | | | | | | | |  | | | | | | | | | | |  | | | | | | | | |  |  |
|  | Log restricted-likelihood = -6.8495749 | | | | | | | | | Prob > Chi2 = 0.0000 | | | | | | | | | | | | | | |  | | | | | | | | | |  | | | | | | | | | | |  | | | | | | | | | |  | | | | | | | | | | |  | | | | | | | | |  |  |
|  | **Fixed effects** | | | | | | Coef. | | | | | | | | SE | | | | | | | | z | | | | | | | p value | | | | | | | | | | 95% CI (Coef.) | | | | | | | | | | | | | | | | | | | | | | | | |  | | | | | | | | | | | | |
|  | | MRC Score | | |  | | | -0.0202391 | | | | | | | | 0.0044915 | | | | | | | | -4.51 | | | | | | | 0 | | | | | | | | | | -0.0290423 | | | | | | | | | | | | | -0.011436 | | | | | | | | | | | |  | | | | | | | | | | | |
|  | | Age | | |  | | | -0.0042519 | | | | | | | | 0.0045535 | | | | | | | | -0.93 | | | | | | | 0.35 | | | | | | | | | | -0.0131767 | | | | | | | | | | | | | 0.0046728 | | | | | | | | | | | |  | | | | | | | | | | | |
|  | | *PRNP* codon 129 genotype (relative to MV) effect on intercept | | | | | | | | | | | | | | | | | | | | | | | | | | | | |  | | | | | | | | | |  | | | | | | | | | | | | |  | | | | | | | | | | | |  | | | | | | | | | | | |
|  | |  | | | MM | | | -0.0101468 | | | | | | | | 0.2011561 | | | | | | | | -0.05 | | | | | | | 0.96 | | | | | | | | | | -0.4044056 | | | | | | | | | | | | | 0.3841119 | | | | | | | | | | | |  | | | | | | | | | | | |
|  | |  | | | VV | | | 0.1482622 | | | | | | | | 0.16386 | | | | | | | | 0.9 | | | | | | | 0.366 | | | | | | | | | | -0.1728975 | | | | | | | | | | | | | 0.4694219 | | | | | | | | | | | |  | | | | | | | | | | | |
|  | | *PRNP* codon 129 genotype (relative to MV) effect on slope (log-tau vs time) | | | | | | | | | | | | | | | | | | | | | | | | | | | | | | | | | | | | | | |  | | | | | | | | | | | | |  | | | | | | | | | | | |  | | | | | | | | | | | |
|  | | 1 | | | MM | | | -0.0173681 | | | | | | | | 0.0188064 | | | | | | | | -0.92 | | | | | | | 0.356 | | | | | | | | | | -0.0542281 | | | | | | | | | | | | | 0.0194918 | | | | | | | | | | | |  | | | | | | | | | | | |
|  | | 3 | | | VV | | | -0.0041224 | | | | | | | | 0.0158515 | | | | | | | | -0.26 | | | | | | | 0.795 | | | | | | | | | | -0.0351907 | | | | | | | | | | | | | 0.0269459 | | | | | | | | | | | |  | | | | | | | | | | | |
|  | | Constant | | |  | | | 2.566831 | | | | | | | | 0.2914613 | | | | | | | | 8.81 | | | | | | | 0 | | | | | | | | | | 1.995578 | | | | | | | | | | | | | 3.138085 | | | | | | | | | | | |  | | | | | | | | | | | |
|  | | | **Random effects (by individual)** | | | | | | | | | | | | | |  | | | | | | | | |  | | | | | | | | | | | | |  | | | | | | | | | | | | | | | |  | | |  | | | | | | | | | |  | | | | | | | | | |
|  | | | Parameters | | |  | | | Estimate | | | | | | | | SE | | | | | | | | | 95% CI (Estimate) | | | | | | | | | | | | | | | | | | | | | | | | | | | | |  | | |  | | | | | | | | | |  | | | | | | | | | |
|  | | | SD (Constant) | | | | | | 0.2487093 | | | | | | | | 0.0343095 | | | | | | | | | 0.1897879 | | | | | | | | | | | | | 0.3259234 | | | | | | | | | | | | | | | |  | | |  | | | | | | | | | |  | | | | | | | | | |
|  | | | SD (MRC Score) | | | | | | 1.16E-13 | | | | | | | | . | | | | | | | | | . | | | | | | | | | | | | | . | | | | | | | | | | | | | | | |  | | |  | | | | | | | | | |  | | | | | | | | | |
|  | | | Residuals: | | | Independent | | | | | | | | | | |  | | | | | | | | |  | | | | | | | | | | | | |  | | | | | | | | | | | | | | | |  | | |  | | | | | | | | | |  | | | | | | | | | |
|  | | | By PRNP codon 129 genotype | | | | | | | | | | | | | |  | | | | | | | | |  | | | | | | | | | | | | |  | | | | | | | | | | | | | | | |  | | |  | | | | | | | | | |  | | | | | | | | | |
|  | | | MM | | | sd(e) | | | 0.1982812 | | | | | | | | 0.0703761 | | | | | | | | | 0.0988926 | | | | | | | | | | | | | 0.3975566 | | | | | | | | | | | | | | | |  | | |  | | | | | | | | | |  | | | | | | | | | |
|  | | | MV | | | sd(e) | | | 0.1205778 | | | | | | | | 0.0141177 | | | | | | | | | 0.0958529 | | | | | | | | | | | | | 0.1516802 | | | | | | | | | | | | | | | |  | | |  | | | | | | | | | |  | | | | | | | | | |
|  | | | VV | | | sd(e) | | | 0.1938962 | | | | | | | | 0.0475099 | | | | | | | | | 0.1199504 | | | | | | | | | | | | | 0.3134274 | | | | | | | | | | | | | | | |  | | |  | | | | | | | | | |  | | | | | | | | | |
|  | | | LR test vs. linear regression: chi2(4) = 50.42 | | | | | | | | | | | | | | | | | | | | | | | Prob > Chi2 = 0.0000 | | | | | | | | | | | | | | | | | | | | | | | | | | | | |  | | |  | | | | | | | | | |  | | | | | | | | | |

# Supplementary Table 4. PrP^Sc^ type and plasma tau and NfL levels

A subset of sCJD patients in the study proceeded to post mortem examination and PrP^Sc^ types were determined as described previously^2^. The London classification^3,4^ is different from a widely used consensus classification^5^, in that Parchi type 1 and 2 are equivalent to London types 2 and 3 in that the London system divides Parchi type 1 into two types, 1 and 2. We found some evidence that plasma tau levels are higher in London MM type 2 (Parchi type 1) in keeping with the typical faster progression of this type.

| **Chart Label** | **c129** | **PrP^Sc^ Type (London)** | **PrP^Sc^ Type (Parchi)** | **n** | **Median Tau** | **Mann-Whitney U Test** | | **Median NfL** | **Mann-Whitney U Test** | |
| --- | --- | --- | --- | --- | --- | --- | --- | --- | --- | --- |
| MM-2 (n=13) | MM | 2 | 1 | 13 | 34.00 | **Z** | 2.571 | 118.26 | **Z** | 0.833 |
| MM-3 (n=8) | MM | 3 | 2 | 8 | 10.38 | **p-value** | 0.010 | 176.84 | **p-value** | 0.407 |
|  |  |  |  |  |  |  |  |  |  |  |
| MV-2 (n=7) | MV | 2 | 1 | 7 | 1.84 | **Z** | 0.635 | 123.06 | **Z** | 0.033 |
| MV-3 (n=16) | MV | 3 | 2 | 16 | 3.12 | **p-value** | 0.529 | 119.05 | **p-value** | 0.976 |
|  |  |  |  |  |  |  |  |  |  |  |
| VV-2 (n=1) | VV | 2 | 1 | 1 | 6.34 | n/a | | 760.84 | n/a | |
| VV-3 (n=8) | VV | 3 | 2 | 8 | 5.46 |  |  | 178.28 |  |  |

# Supplementary Figure 1. Distribution of rate of change in plasma tau and NfL between all serial sample pairs in sCJD

Plasma NfL tended to increase over time, and with increasing levels of functional impairment: in 63 serial sample pairs from 44 individuals, the median rate of change in plasma NfL was +2.88% per week (IQR +0.58% to 7.32%). Plasma tau also tended to increase over time, although this was more variable: in 60 sample pairs from 41 individuals the median rate of change was +2.37% per week (IQR -1.3% to +15%).

**
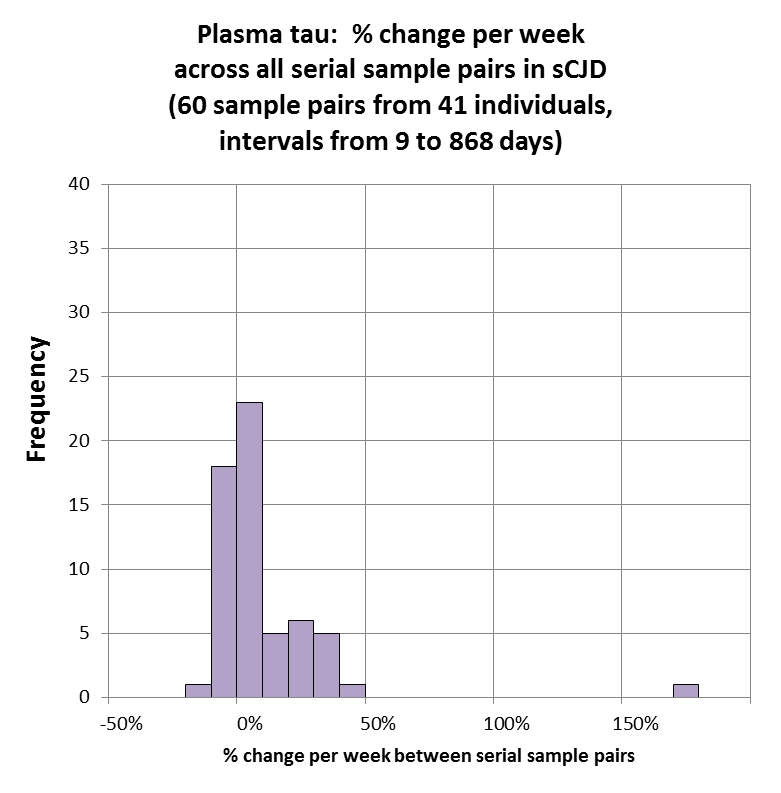

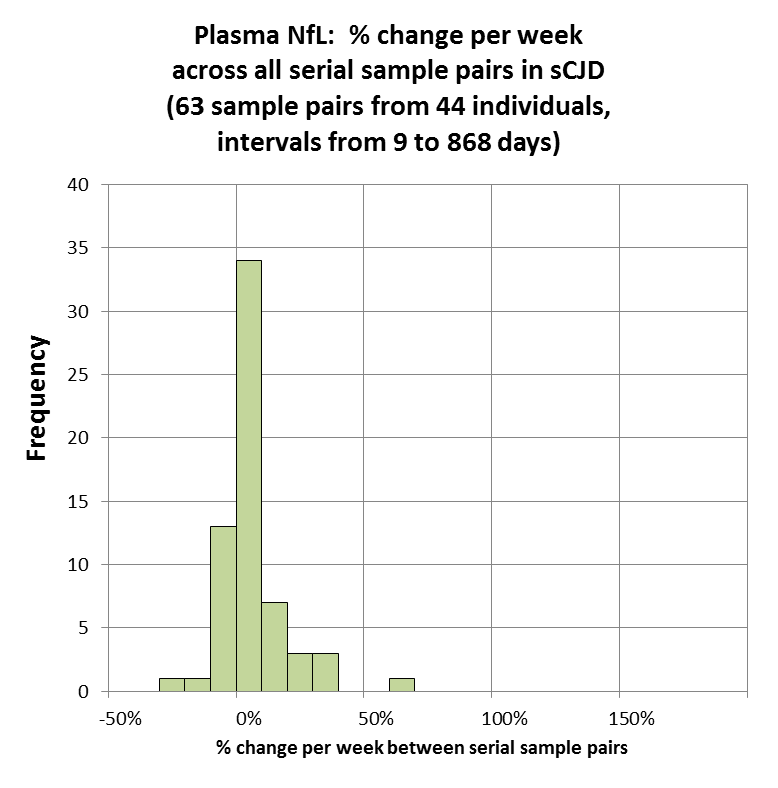
**

# **Supplementary Figure 2. Comparing plasma tau and plasma NfL (both shown on log scales) between *PRNP* mutations in symptomatic IPD**

We used the earliest available sample for all *PRNP* mutation carriers with symptomatic IPD. Samples from individuals with symptomatic IPD due to 12 different *PRNP* mutations were included in the study (P102L (26), 6OPRI (19), E200K (10), 5OPRI (7), D178N (6), A117V (5), 4OPRI (3), Y163X (3), E196K (1), P105L (1), Q212P (1), V210I (1)). Including a single sample (earliest available) for each individual, we compared plasma tau and NfL between mutations. Mutations typically causing a rapid CJD-like disease phenotype (E200K, 4OPRI, E196K, V210I) were associated with very high concentrations of both tau and NfL in plasma, similar to those seen in sCJD, while the D178N mutation (typically causing either a CJD-like or fatal familial insomnia phenotype) gave slightly lower concentrations. Mutations typically causing Gerstmann-Straussler-Scheinker syndrome (P102L, A117V, P105L) seemed to result in a modest increase in tau and a relatively marked (though more variable) increase in NfL. Mutations typically causing a relatively slow cognitive decline (5OPRI, 6OPRI) produced more modest elevations, similar to those seen in the AD and FTD groups, with whom their clinical presentations may overlap.

Supplementary Figure 3. Relationship of plasma tau and NfL to number of days from death in symptomatic IPD, including both cross-sectional and longitudinal datasets

Red continuous, dashed and dotted lines show 50^th^, 75^th^ and 90^th^ centiles of healthy controls respectively. Blue lines show the linear mixed effects model fits for each individual in the longitudinal dataset. Changes in plasma biomarkers with disease progression in IPD were also studied, using similar methods to those described above for sCJD. For plasma NfL, both the cross-sectional (baseline) data and the longitudinal data showed that concentration tends to increase as IPD progresses, and this was supported by linear mixed modelling (method as described for sCJD, but with *PRNP* mutation included as a covariate in place of *PRNP* codon 129).

# **Supplementary Figure 4. Plasma NfL concentrations plotted against age at time of sampling for controls and asymptomatic at-risk individuals**

Plasma NfL concentrations plotted against age at time of sampling for all samples taken from asymptomatic individuals, including Healthy Controls (n = 34) and PRNP mutation carriers (n = 32). “Slow IPD”: P102L x 5, 5OPRI x 1, 6OPRI x 1. “Fast IPD”: E200K x 1, D178N x 1.  Samples taken within 2 years of a known date of symptom onset (occurring during follow up) are highlighted in red, and the time delay from last asymptomatic sample until time of symptom onset is shown. a, b these samples with high NfL levels were from participants lost to follow up within two years of the sample being taken.

# **Supplementary Figure 5. Relationship of plasma tau and NfL to number of days from death in iCJD and vCJD, including both cross-sectional and longitudinal datasets**

Circles indicate samples from patients with vCJD, diamonds indicate samples from patients with iCJD. Red continuous, dashed and dotted lines show 50^th^, 75^th^ and 90^th^ centiles of healthy controls respectively. As for sCJD, the differences in plasma tau and NfL between diagnostic groups are summarised in Table 1, and in the box plots in Figure 1.

# References

1. Mead S, Burnell M, Lowe J, et al. Clinical Trial Simulations Based on Genetic Stratification and the Natural History of a Functional Outcome Measure in Creutzfeldt-Jakob Disease. *JAMA Neurol* 2016; **73**(4): 447-55.

2. Wadsworth JDF, Adamson G, Joiner S, et al. Methods for Molecular Diagnosis of Human Prion Disease. *Methods Mol Biol* 2017; **1658**: 311-46.

3. Wadsworth JD, Hill AF, Beck JA, Collinge J. Molecular and clinical classification of human prion disease. *Br Med Bull* 2003; **66**: 241-54.

4. Hill AF, Joiner S, Wadsworth JD, et al. Molecular classification of sporadic Creutzfeldt-Jakob disease. *Brain* 2003; **126**(Pt 6): 1333-46.

5. Parchi P, de Boni L, Saverioni D, et al. Consensus classification of human prion disease histotypes allows reliable identification of molecular subtypes: an inter-rater study among surveillance centres in Europe and USA. *Acta Neuropathol* 2012; **124**(4): 517-29.
